# Effect of Porous Structure on the Microwave Absorption Capacity of Soft Magnetic Connecting Network Ni/Al_2_O_3_/Ni Film

**DOI:** 10.3390/ma13071764

**Published:** 2020-04-09

**Authors:** Hu Wei, Li Cheng, Dmitry Shchukin

**Affiliations:** 1 College of Materials Science & Technology, Nanjing University of Aeronautics and Astronautics, Nanjing 210016, China; licheng@nuaa.edu.cn; 2 Stephenson Institute for Renewable Energy, Department of Chemistry, University of Liverpool, Chadwick Building, Peach Street, Liverpool L69 7ZF, UK; d.shchukin@liverpool.ac.uk

**Keywords:** connected network Ni/Al_2_O_3_/Ni, soft magnetic properties, microwave absorption

## Abstract

Microwave radar absorbing materials have been the focus of the radar stealth research field. In this study, ceramic structured porous honeycomb-like Al_2_O_3_ film was prepared by anodic oxidation, and an Ni layer was deposited on the Al_2_O_3_ film via electrodeposition in a neutral environment to form a flower- and grain-like structure in a three-dimensional (3D) network Ni/Al_2_O_3_/Ni film. The films both have a through-hole internal structure, soft magnetic properties, and absorb microwaves. The dielectric loss values of two films were little changed, and the maximum microwave absorption values of flower- and grain-like Ni/Al_2_O_3_/Ni film were −45.3 and −31.05 dB with relatively wide effective bandwidths, respectively. The porous ceramic structure Al_2_O_3_ interlayer prevented the reunion of Ni and isolated the eddy current to improve the microwave absorption properties. The material presented in our paper has good microwave absorption performance with a thin thickness, which indicates the potential for lightweight and efficient microwave absorption applications.

## 1. Introduction

Magnetic overlapping multilayer films formed by alternately depositing ferromagnetic materials on an insulating material substrate have good chemical stability, a tunnel magnetoresistance effect, and a microwave absorption function [1]. The exploration of this artificial nanostructure has broadened the research field of magnetic transmission theory [2,3]. According to the giant magnetoresistance (GMR) in thin film structures composed of alternating ferromagnetic and nonmagnetic layers [4], as adjacent magnetic layers are always separated by non-magnetic layers, the coupling pattern of magnetic moments of adjacent layers usually changes from anti-parallel or disordered arrangement to parallel arrangement under the action of an external magnetic field, which may be caused by a non-magnetic isolation structure or the change in an internal magnetic field. To obtain lightweight, efficient, and stealthy microwave absorption materials, scientists further studied the interaction between microwaves and this kind of nanocomposite [5]. Kang et al. [6] proved that the soft magnetic properties of porous carbon nanotubes significantly improved after filling with ferromagnetic metals.

Other research showed that using Al_2_O_3_ as an insulating layer for magnetic particles can improve the microwave stealth ability of magnetic materials [7]. Eriksson et al. [8] designed a new type of ring parallel plate resonator with excellent microwave absorption performance using the transparent property of the modulated Cu/SiO_2_/Cu multilayer film. Wei et al. prepared an Fe_3_Al composite using a mechanochemical method and coated Al_2_O_3_ as an insulating layer on the surface of Fe_3_Al. The reflectivity value of this Fe_3_Al/Al_2_O_3_/Fe_3_Al absorbing coating was lower than −20 dB in the range from 7.2 to 17.4 GHz when the thickness of the Al_2_O_3_ coating was 1.5–2.5 mm [9]. In general, the non-magnetic porous structure of Al_2_O_3_ provides nucleation points for magnetic particles so that the magnetic particles can be highly dispersed. As the material dielectric loss and magnetic loss enhance the microwave attenuation ability, metals can be prepared with Cr, Ni, or their alloys; while ceramic matrices can also be prepared with sapphire, quartz, BN, and BeO [10].

Magnetic metal nanoparticles easily agglomerate; the ceramic Al_2_O_3_ network structure acts as an insulator layer inside the metal magnetic medium, which also blocks the eddy current effect and reduces electromagnetic loss. In this paper, a 3D connected network structured Ni/Al_2_O_3_/Ni film was constructed on the ceramic structure Al_2_O_3_ film via electrodeposition. The Ni/Al_2_O_3_/Ni film has good soft magnetism and microwave absorption properties within a frequency range of 2 to 18 GHz. The connected network structure can considerably reduce the density, optimize the impedance matching of films, and avoid the agglomeration of the nanomaterials. Unlike traditional vertical one-dimensional (1D) channel alumina, the 3D staggered pipe structure inside ceramic structured alumina film better isolates the dispersed metal particles and reduces the eddy current effect between the particles [11].

## 2. Materials and Methods

High-purity aluminum foil (Al 99.99% with a thickness of 200 μm) was cut into 6 × 6 cm^2^ rectangle samples and annealed in a tube-type vacuum furnace at 500 °C for 3 h in a nitrogen atmosphere. Then, the substrate surface was cleaned in absolute ethyl alcohol under bath ultrasonic treatment for 20 min. Afterward, aluminum samples were introduced into NaOH 1 M to clear the surface oxide for 1 min at 40 °C and HNO_3_ 1 M (to neutralize OH^–^) for 2 min at room temperature (25 °C). Finally, the samples were washed with distilled water. All the chemicals were of analytical grade and used as purchased. The electrolyte was an equal volume mixture of aqueous 5 wt % phosphoric acid and 3.6 wt % oxalic acid, with an aluminum sheet as the anode and tinplate as the cathode. The samples were pretreated for 2 h under the 50 V DC voltage at 10 °C, then taken out, washed with distilled water, and soaked into mixed acid (phosphoric acid containing 6 wt % and chromate 1.8 wt %) for 3 h at 60 °C to eliminate the irregular oxide layer on the pure Al substrate. The second anodic oxidation was carried out for 2 h under the same conditions as the first anodic oxidation, and we obtained an alumina film with a porous ceramic structure.

The samples were used as the working electrode and a platinum electrode as the counter electrode; an electrochemical workstation (CHI 750C, Chenhua, Shanghai, China) was used. The Ni nanoparticles were plated by cyclic voltammetry from a 1 M NiSO_4_ solution with pH around 7.2 for 6000 s. Pure (99.99%) Al_2_O_3_ powder was mixed with the Ni sample using the hydrothermal method (100 °C) for 2 h on ceramic structured alumina film, followed by annealing at 1000 °C for 5 h.

The samples’ morphology and composition were analyzed by scanning electron microscopy (SEM, Hitachi S-4800, Tokyo, Japan) and energy-dispersive X-ray spectrometer (EDS). The crystal composition of the samples was measured by X-ray diffraction (XRD, scanned from 10° to 80° with a step size of 0.02° and scanning speed 2°/min) with a Cu-Kα radiation source (λ = 0.154056 nm), and the spectrum was analyzed using Jade 6.0 software (Jade Company, Christchurch, New Zealand). The static magnetic properties of the samples at room temperature were measured using a comprehensive physical properties testing system (PPMS-9, Quantum Design, San Diego, CA, USA) and a vibrating sample magnetometer (VSM, Lakeshore 7407, Columbus, OH, USA). The measured temperature was 25 °C and the maximum applied magnetic field strength was 10 kOe. Using liquid nitrogen (77 K) as the absorbent, the specific surface area of the compound was measured using an ASAP 2020 4.01 version instrument (Micromeritics, Norcross, GA, USA), the details of which were: ambient temperature: 22.00 °C; analysis bath temperature: −195.792 °C; equilibration interval: 10 s; warm free space: 27.9249 cm³; and cold free space: 86.2767 cm³. The electromagnetic microwave properties were measured using the reflection transmission network method using the Microwave Network Analyzer (MNA, HP 8722ES, Xiamen, China) in the frequency range of 2 to 18 GHz. The dielectric testing fixture was tested using sweeping frequency sweep (Agilent 6453A, Xiamen, China), and the test sample was shaped as a disc with a radius of 5.5 cm and a thickness of 1.7 cm. The microwave absorbing properties of the pure Ni/Al_2_O_3_ powder sample was tested after being mixed with 15 vol % paraffin wax filling.

## 3. Results

**Figure 1:**
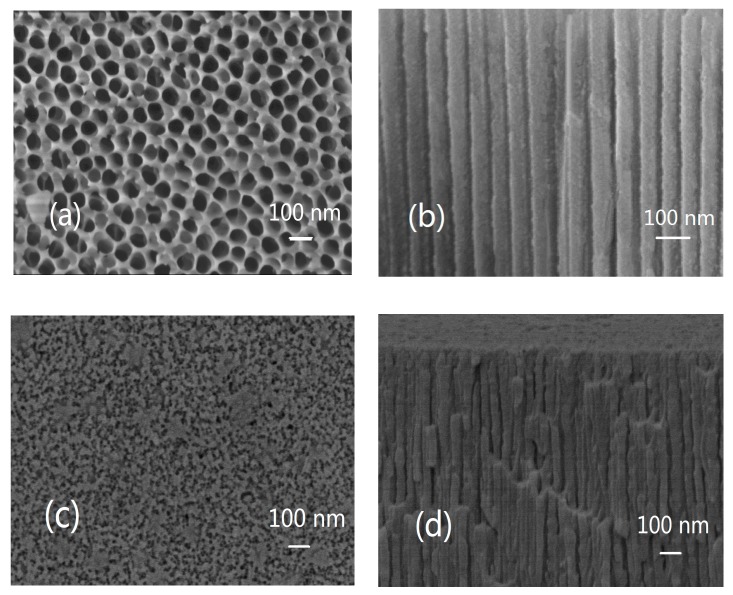

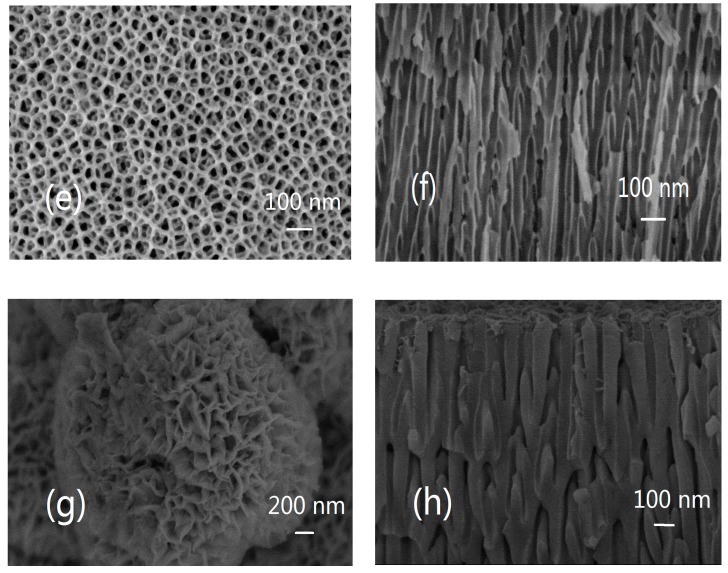
SEM images of the top view and the side view of the traditional alumina film (**a**,**b**) and Ni deposited on traditional straight pass alumina film (**c**,**d**); the ceramic structure alumina film (**e**,**f**) and Ni deposited on the ceramic structure alumina film (**g**,**h**) by the electrodeposition method.

The SEM images of the alumina surface after anodic oxidation demonstrated a pore size of around 100 nm and an interconnection between the pores. This overlapped porous structure is different from the traditional 1D vertical pipe alumina membrane (Figure 1a). The traditional alumina has regular vertical tubes (Figure 1b). Crystals accumulated and grew into many grain-like particles on the film surface, and a small amount of grain-like Ni/Al_2_O_3_/Ni appeared from a holistic perspective (Figure 1c). The surface of Ni/Al_2_O_3_/Ni prepared on the surface of traditional straight pass alumina only formed vertical parallel nickel-plated pipes, in which Ni^2+^ in the solution was reduced and gradually deposited inside alumina pipelines (Figure 1d). From the top view, the ceramic structure alumina membrane with an internal communication network is composed of many crossing pipelines (Figure 1e) and the interior has a large number of 3D passageways, which resemble many overlapping nets from above. After being oxidized excessively, the tube walls melt and irregular faults form in the vertical direction inside the alumina (Figure 1f) Ni particles deposited outward along the internal channels of the alumina accumulated on the film surface like porous petals. The porous structure was maintained and resembled Ni laces around those holes (Figure 1g). Overall, the deposited Ni piled up like flowers with increasing deposition time (Figure 1h). Microscopic examination indicated that metallic Ni was uniformly deposited on the alumina nanotube.

**Figure 2:**
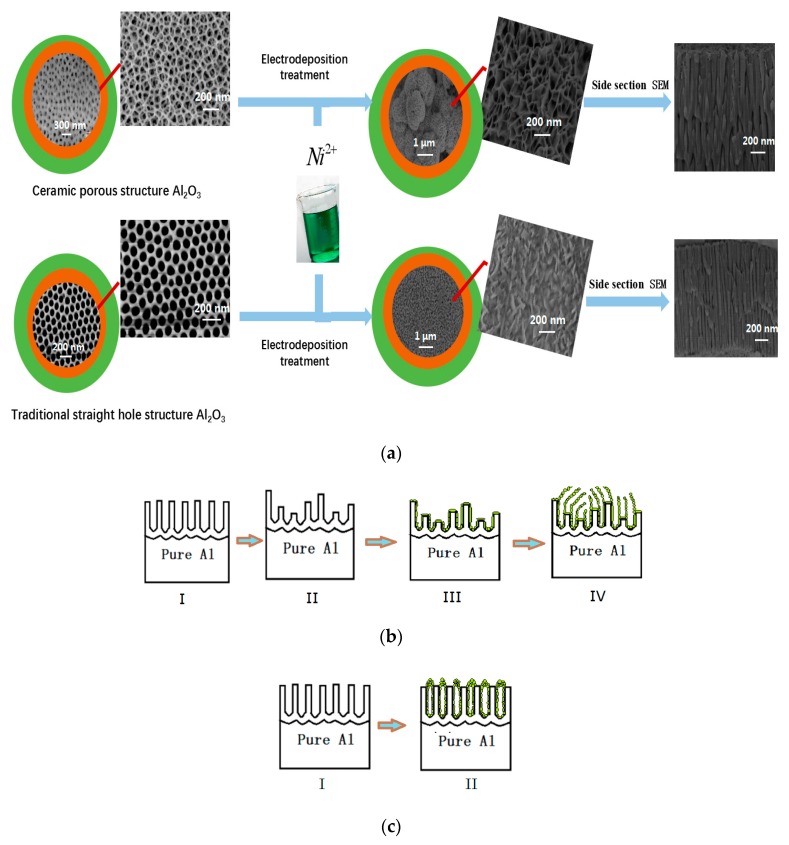
(**a**) Diagram of preparation process of Ni/Al_2_O_3_/Ni composite films; (**b**,**c**) growth mechanism of two kinds of Ni/Al_2_O_3_/Ni composite films.

The SEM images reveal the formation mechanism of the Ni/Al_2_O_3_/Ni composite film (Figure 2a). Ni particles were deposited along alumina nanotubes, which then grew out of the alumina film surface. Ni formed a branched hollow tubular deposit along the tube wall of the ceramic structure Al_2_O_3_ film instead of being closely packed. Certain angles in the ceramic structure Al_2_O_3_ film provide horizontal force, and due to the space limitation, the Ni petals do not grow indefinitely but form porous spherical flowers and accumulate together into clusters. The Ni/Al_2_O_3_/Ni composite film still has a 3D connected network structure in the inner cavity. The pore’s size can be designed according to specified criteria so that they can be filled with microwave absorbing to conveniently provide expected material properties [12]. The 3D connected network inside Al_2_O_3_ provides effective isolation and prevents nickel agglomeration.

The flower-like Ni/Al_2_O_3_/Ni film was prepared on the surface of the ceramic structure alumina, whereas the grain-like structural membrane was prepared on the surface of traditional straight pass alumina via electrodeposition. The Ni particles grew along the tube walls inside the alumina and formed a flower-like structure (Figure 2b). The reason for the synthesized grain-like Ni surface may be that the deposited Ni grew along the tube walls inside traditional straight pass alumina and formed a vertical pipe structure, which resulted in Ni accumulation on the surface forming grain-like particles (Figure 2c). The tube walls inside the alumina effectively isolated the agglomeration of the Ni layer, which would strongly reduce the magnetic field consumption caused by the eddy current effect.

**Figure 3:**
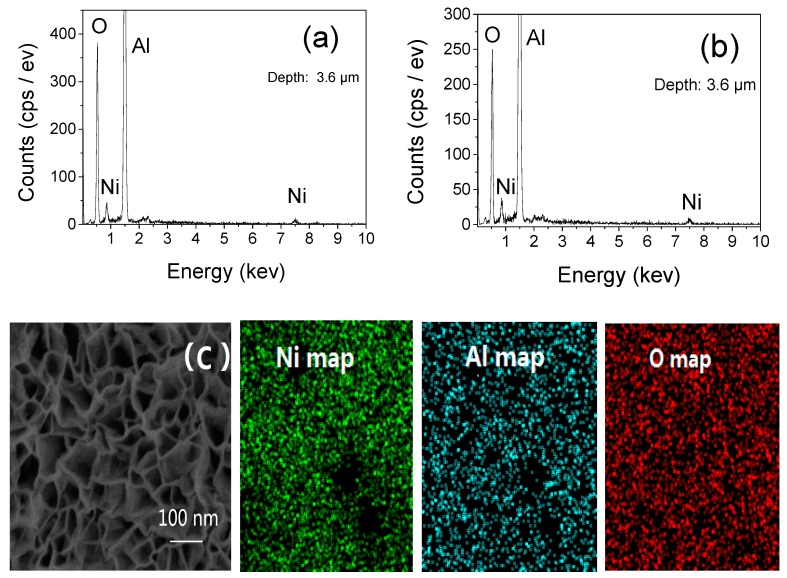
EDS pattern of the as-prepared (**a**) flower-like and (**b**) grain-like Ni/Al_2_O_3_/Ni film and (**c**) enlarged SEM image of the flower-like Ni/Al_2_O_3_/Ni film and the corresponding mapping of Ni, Al, and O.

According to EDS (Figure 3), the membrane components were Ni, Al, and O; no other electrolyte material was found. Table 1 and Table 2 correspond to the component content data of Figure 3a,b respectively. From Table 1 and Table 2, the Ni content was 5 wt %, demonstrating a thinner coating layer than the alumina sheet, although the Ni content was not high compared with the aluminum oxide film prepared. A series of experimental results below showed that this Ni content is sufficient to produce ferromagnetism and microwave absorption, and the hollow structure and crystal form of Ni particles play the most important roles in creating microwave absorption characteristics. The Ni layer is much thinner than the alumina sheet, a large amount of Ni particles piled up like many small protrusions on the alumina surface. However, the Ni content in the flower-like Ni/Al_2_O_3_/Ni film (Figure 3a) was slightly higher than in the grain-like Ni/Al_2_O_3_/Ni film (Figure 3a) because the flower-like Ni produced by electrochemical deposition extended to the external 3D space to increase the space for Ni growth and increase the content. According to the uniform high definition imaging for the entire observation area of the Ni map, we deduced that flower-like Ni/Al_2_O/Ni_3_ has homogeneous growth on the micrometer scale (Figure 3c).

**Figure 4:**
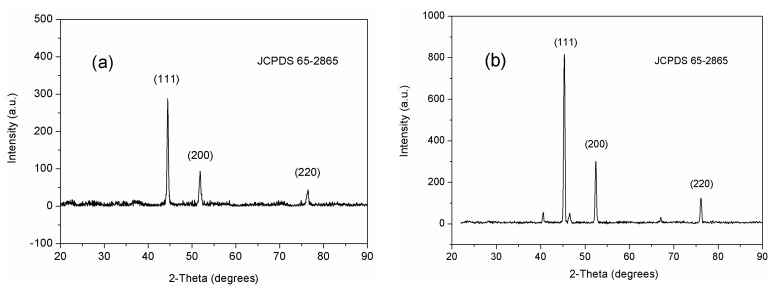
Small-angle XRD diffraction spectrum of the (**a**) flower-like Ni/Al_2_O_3_/Ni film and (**b**) grain-like Ni/Al_2_O_3_/Ni film.

The ceramic-based composite with flower-like Ni deposited on Al_2_O_3_ nanotubes was fabricated using electrochemistry, and the small-angle XRD diffraction spectrum showed crystals with a face-centered cubic structure and three characteristic peaks at 44.49°, 51.85°, and 76.56° in the lattice plane index (Figure 4a). The XRD diffraction peaks for the grain-like Ni/Al_2_O_3_/Ni film were located at 45.05°, 52.53°, and 76.86°, corresponding to the Ni crystal face (111), (200) and (220), respectively (Figure 4b), which matches the nickel pattern according to the international standard card JCPDS: 65-2865. The 40.22°, 46.38°, 67.49° diffraction peak was related to the γ-Al_2_O_3_ basement, because the Ni deposition layer on the surface of the sample is relatively thin, the space size of granular Ni particles is much smaller than that of flower-like Ni (see Figure 1), and the depth of diffraction of XRD by small-angle diffraction is easier to hit the Al_2_O_3_ substrate. The average size of the grains can be calculated using the Scherrer formula [13]:

(1) $$D=\frac{0.89\lambda }{\beta \cos\theta }$$

where D is the average grain size, θ is the second diffraction angle, λ is the wavelength of the X-ray (λ = 1.5406 Å), and β is the integral half-width. Because the characteristic peaks of the Al and Al_2_O_3_ matrix are the extraneous peaks of the electrodes, they can be ignored when calculating the average size of the grains using the Scherrer formula. The calculated average grain size of Ni was about 9.7 ± 0.1 nm. The Curie temperature of magnetic metal absorbing material is high, and magnetic metal is prone to the eddy current phenomenon, which reduces its saturation magnetization and weakens the absorption of the microwave. The diffraction peaks of Ni widened, apparently due to its nanoscale effect, and the peaks were sharp, indicating good crystallinity. No other impurity peaks, such as NiO or Ni(OH)_2_, were detected. The shape of the Ni crystals is mainly affected by the relative Ni growth rate in different Al_2_O_3_ channel directions; rugged channels grow into flower-like Ni, whereas straight channels grow into grain-like Ni [14].

**Figure 5:**
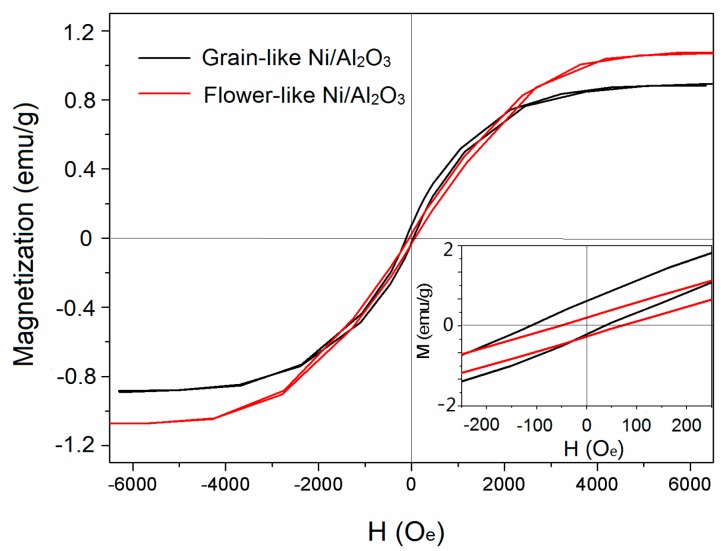
Hysteresis loop curves of flower-like and grain-like Ni/Al_2_O_3_/Ni films.

The hysteresis loop curves of flower- and grain-like Ni/Al_2_O_3_/Ni were measured by VSM at room temperature (25 °C), as shown in Figure 5. The two samples revealed the ferromagnetic properties of the coercivity (Hc), the saturation magnetization (Ms), and the residual magnetization (Mr) values of the flower-like Ni/Al_2_O_3_/Ni were 110 Oe, 1.09 emu/g (1 emu/g = 1 Am^2^/kg), and 0.376 emu/g, respectively; those of the grain-like Ni/Al_2_O_3_/Ni were 105 Oe, 0.849 emμ/g, and 0.25 emμ/g, respectively. However, the intrinsic magnetic properties with high saturation magnetization (Ms) and low coercivity (Hc), which bring a good permeability and magnetic loss and further contribute to the microwave absorption. The two samples’ saturation magnetization were very close [15]. However, compared with the Hc value of pure Ni (0.7 Oe) [16], the coercivity value of the grain-like Ni/Al_2_O_3_/Ni is a little lower than that of flower-like Ni/Al_2_O_3_/Ni. This phenomenon may be due to the increased content of Ni nanoparticles; the reduction in particle size synergistically strengthens the magnetization reversal mechanism [17,18]. Pang et al. [19] reported that the electrons’ spin state and size effect of Ni nanoparticles are the main factors determining their magnetic properties.

The loops of two samples in the direction of the X-axis both showed a narrow rectangular shape, almost coinciding and crossing diagonally in the direction of the X-axis. Comparing the hysteresis loops on the Y with the X-axis, the samples had a static in-plane uniaxial anisotropy field (H_k-stat_) [20]. The exchange coupling effects of multilayer samples include additional interlayer coupling effects when the layer thickness is suitable. According to the cave model proposed by Grunberg et al., when two ferromagnetic layers are separated by a nanometer insulation layer and the thickness of the insulation layer is relatively thin (generally does not exceed 2 nm), the ferromagnetic layer can be completely isolated. The ferromagnetic Ni layers on both sides of the whole walls of Al_2_O_3_ are inclined to be arranged in parallel, so interlayer exchange coupling can be easily obtained in thin films under an externally induced magnetic field [21,22]. Based on the XRD diffraction spectrum and hysteresis loop curves, the coupling action among Ni/Al_2_O_3_/Ni particles can overcome the effect of thermal disturbance and form a continuous magnetic structure with the size of a single magnetic particle.

**Figure 6:**
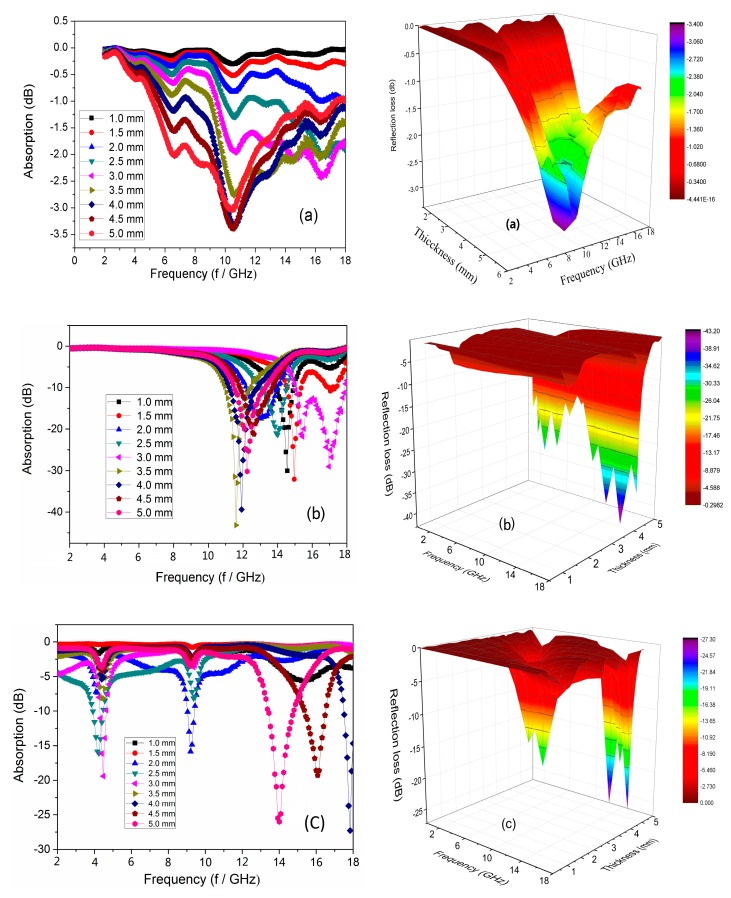
The microwave absorbing (RL) curves with different thicknesses in the same sample of a frequency range of 2 to 18 GHz (on the left) and the corresponding 3D depth maps (on the right). Samples of (**a**) pure Ni/Al_2_O_3_ composites, (**b**) flower-like Ni/Al_2_O_3_/Ni, and (**c**) grain-like Ni/Al_2_O_3_/Ni prepared on the surface of traditional straight pass alumina. All samples have the same thickness of the test.

Of the total energy of microwave absorbed, −10 dB is equivalent to 90% and −20 dB is equivalent to 99% [23]. The RL_max_ of pure Ni/Al_2_O_3_ powder could only reach −3.4 dB, and could not achieve the effective bandwidth (Figure 6a). When the thickness of flower-like Ni/Al_2_O_3_/Ni sample is 3.5 mm and the effective absorption bandwidth (RL ≤ −10 dB) is 7.1 GHz, the microwave maximum reflection loss (RL_max_) is −45.3 dB at 11.4 GHz (Figure 6b). The RL_max_ of grain-like Ni/Al_2_O/Ni_3_ reaches −28.2 dB corresponding to a thickness of 4.5 mm at 12.92 GHz, and effective absorption bandwidth is about 5.5 GHz (Figure 6c). The RL_max_ and effective bandwidth of the flower-like Ni/Al_2_O_3_/Ni sample is 17.1 dB and 1.6 GHz higher than that of the grain-like Ni/Al_2_O_3_/Ni sample, correspondingly. The flower-like Ni/Al_2_O_3_/Ni has more absorption capacity than the grain-like Ni/Al_2_O_3_/Ni because of ceramics porous structure inside flower-like Ni/Al_2_O_3_/Ni help provide isolation of Ni particles agglomeration, especially at the absorption bandwidth of the maximum absorbed microwave intensity. To some extent, the porous ceramic structure improves the microwave absorption performance of Ni/Al_2_O_3_/Ni, enhancing the internal refraction absorption and the dielectric polarization of Ni, which promotes the attenuation of microwaves. The main function of the porous structure of Al_2_O_3_ prepared in this study is the promotion of the growth of porous nickel, providing a support function similar to a skeleton, which is conducive to the formation of magnetic crystals by Ni particles. The flower- and grain-like Ni/Al_2_O_3_/Ni samples have better microwave absorption properties than that pure Ni/Al_2_O_3_; hence, we microwave active materials with the multilevel 3D tubular structure could be developed, which would reflect and absorb microwaves many times inside the ceramic Ni/Al_2_O_3_/Ni film [24]. The ceramic flower-like Ni/Al_2_O_3_/Ni has a high aspect ratio, lower density, and lower content of metallic Ni due to the tubular structure compared with pure Ni/Al_2_O_3_ powder. Other metals or alloys, e.g., Co and Fe, could also be deposited into porous ceramic Al_2_O_3_ nanotubes to tune the microwave absorption properties of the porous composites.

**Figure 7:**
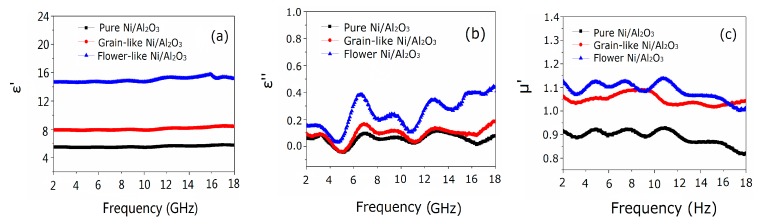

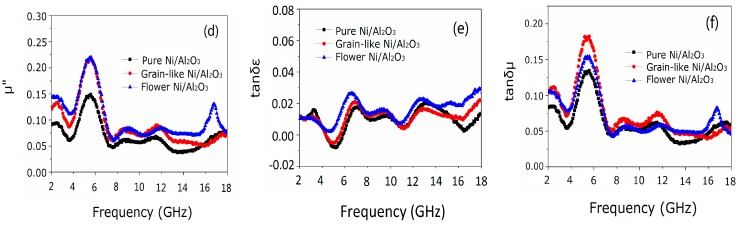
Electromagnetic parameters of three Ni/Al_2_O_3_/Ni samples: real (**a**), imaginary (**b**) part of the complex permittivity; real (**c**), imaginary (**d**) part of the complex permeability and tangent dielectric loss (**e**) and tangent magnetic loss (**f**) in the frequency range of 2–18 GHz.

Figure 7 demonstrates the relationship between the real part and the imaginary part of complex permittivity, real part and imaginary part of complex permeability with the frequency of the flower-like, grain-like, and pure Ni/Al_2_O_3_/Ni samples. The flower-like Ni/Al_2_O_3_/Ni permittivity (real part) ε′ value changes within a narrow range around 19 in the frequency range of 2 to 11 GHz, which is much larger than that of the grain-like sample (Figure 7a). Similarly, the ε′ values of the grain-like Ni/Al_2_O_3_/Ni and pure Ni/Al_2_O_3_ samples were about nine and six, respectively, and the values of the changes in the imaginary part ε″ were in the range of 0.2 to 1.2 (Figure 7b). The ceramics flower-like Ni/Al_2_O_3_/Ni sample was semiconductor-like and a small number of electrons in the core could participate in conduction through the Ni layer. Therefore, we initially established the connected network Ni/Al_2_O_3_/Ni composite material system. Due to the electrical conductivity and alternating microwave-field-induced eddy current in the flower-like Ni, the eddy current can be isolated by an Al_2_O_3_ insulating medium, and the temperature generated by eddy current can be effectively reduced [25]. The real part ε′ of the permittivity increased gradually with the frequency of the alternating microwave field; as the relationship between the complex permittivity’s imaginary part ε″ and the conductivity ε″ ≈ σ/2πfε_0_ is constant (where σ, f, and ε_0_ are the resistivity, the frequency and the dielectric constant of free space, respectively.), the imaginary part ε″ of the Ni/Al_2_O_3_/Ni nanoparticles increases with increasing frequency [26]. Generally speaking, the magnetic loss mainly comes from magnetization vector rotation, hysteresis loss, magnetic domain wall resonance, natural resonance, and eddy current loss. Among them, the magnetization vector rotation mainly occurs under the action of a strong magnetic field, the lag loss of soft magnetic materials is very limited, and the domain wall resonance mainly occurs in the range of 1–100 MHz, so the electromagnetic wave loss of sample mainly comes from natural resonance and eddy current loss. However, the magnitude of eddy current loss is related to the thickness (d) and electrical conductivity (σ) of the absorber, which can be expressed by the following formula [27]:

(2) $${\mu_{r}}^{\prime \prime }=3\pi \mu_{0}{\left(\mu^{\prime }\right)}^{2}\sigma d^{2}f$$

In Equation (2), d is the thickness of the absorber, f is the frequency of the microwave, μ_0_ is permeability in a vacuum. If the magnetic loss of samples all comes from eddy current loss, then the value is constant in the measured frequency range [28]. As shown in Figure 7, $\mu^{\prime \prime }{\left(\mu^{\prime }\right)}^{-2}f^{-1}$ is not a constant as the frequency increases, therefore, the microwave loss of the sample mainly comes from the natural resonance and occurs in ranges of 4–8 GHz and 16–18 GHz (Figure 7c,d).

From the composite complex imaginary part of the permeability constant values of Ni/Al_2_O_3_/Ni samples, we observed several peaks in the frequency range of 4–8 GHz. The main reason for these peaks is the quantum size effect of Ni nanoparticles that results in the separation of electron energy levels [29]. The composite has low magnetic conductivity, magnetic loss, and dielectric loss values under an applied magnetic field within 2–18 GHz. The change in the tangent dielectric loss tanδε (tanδε = ε″/ε′) and tangent magnetic loss tanδμ (tanδμ = μ″/μ′) of the grain-like (0.33 and 0.14) Ni/Al_2_O_3_/Ni are greater than that of the flower-like sample (0.29 and 0.11, Figure 7e,f).

α is the electromagnetic attenuation constant and is the main parameter of the microwave attenuation effect of the reaction material, and can be calculated by the following calculation formula:

(3) $$\alpha =C\sqrt{\mu^{\prime }\varepsilon^{\prime }}\cdot \sqrt{\frac{1}{2}\left|\tan\delta_{\varepsilon }\tan\delta_{\mu }-1+\sqrt{1+\tan^{2}\delta_{\varepsilon }+\tan^{2}\delta_{\mu }+\tan^{2}\delta_{\varepsilon }\tan^{2}\delta_{\mu }}\right|}$$

where C is the attenuation coefficient of the material. The dielectric loss angle tangent tanδε (around 0.01) of the three Ni/Al_2_O_3_/Ni samples was much smaller than tanδμ (around 0.1), the electromagnetic attenuation constant is dominated by magnetic attenuation, so the microwave absorbing mechanism of Ni/Al_2_O_3_/Ni mainly depends on magnetic loss.

**Figure 8:**
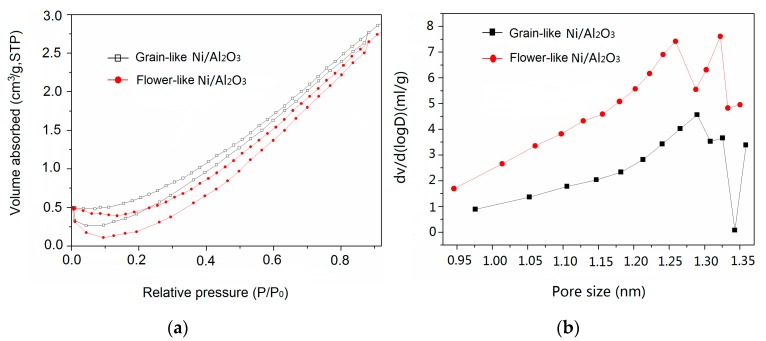
(**a**) Nitrogen adsorption–desorption isotherms and (**b**) pore size distributions of grain-like and flower-like Ni/Al_2_O_3_/Ni film.

Flower-like and granular Ni/Al_2_O_3_/Ni samples both have nanopores inside (Figure 8a), but the composite membrane constructed on the surface of Al_2_O_3_ film with a ceramic structure has a larger pore diameter and better nitrogen adsorption–desorption capacity than that of the traditional Al_2_O_3_ film (Figure 8b). Capillary condensation extends to P/P_0_ indicates that the alumina pores in the composite membrane are completely filled and the structural porosity is high, which is consistent with the results of SEM. According to the effective medium theory, the effective dielectric constant is reduced by dispersion of magnetic particles, and the impedance matching condition can be improved [30]. The large porosity indicates that there are large numbers of porous structures in this membrane, which can promote its microwave absorption capacity. The combination of magnetic characteristics of Ni and the porous structure of the ceramic alumina structure enhances the electromagnetic wave and magnetic absorption properties of the composite porous Ni/Al_2_O_3_/Ni. As a result, it is more beneficial to add other materials that are beneficial to improving microwave absorption or electromagnetic performance through its porous structure, such as graphite, carbon-containing manganese oxide powder, etc. [31]. The dense material is designed as a porous material, which is equivalent to a large number of air cavities. In addition, the density of porous materials can be markedly reduced to meet the requirements of lightweight stealthy materials.

Generally speaking, the parameters of porous materials have a certain relation to the effective permittivity and the reflectivity RL. If the porosity of the porous material is f, the dielectric constant of the skeleton material is ε = ε′ + jε″, the dielectric constant j of the air cavity is 1, and the linear approximation models based on the effective dielectric constant are [32]:

(4) $$\varepsilon_{\varepsilon }=p+\varepsilon \left(1-p\right)=f+\varepsilon^{\prime }\left(1-p\right)+\varepsilon^{\prime \prime }\left(1-p\right)$$

where ε_ε_ is the effective permittivity of porous material. The following formulas according to the effective permittivity ε_ε_ = ε_ε__′_ + jε_ε__″_ [33]:

(5) $$\varepsilon^{\prime }=\varepsilon_{\infty }+\frac{\varepsilon_{s}-\varepsilon_{\infty }}{1+\omega^{2}\tau^{2}}$$

(6) $$\varepsilon^{\prime \prime }=\frac{\sigma }{{\omega \varepsilon }_{0}}+\frac{\varepsilon_{s}-\varepsilon_{\infty }}{1+\omega^{2}\tau^{2}}\omega \tau$$

where ε_∞_ and ε_s_ are the relative permittivity and static permittivity at a high-frequency limit, respectively. When the microwave is perpendicular to the surface of material, the reflectivity RL can be expressed as:

(7) $$RL\left(dB\right)=20\log_{10}\left|\frac{Z_{in}-1}{Z_{in}+1}\right|$$

(8) $$Z_{\mathrm{in}}=\sqrt{\frac{\mu }{\varepsilon }}\tan h\left(\frac{2\pi \mathrm{fdj}\sqrt{\mu \varepsilon }}{c}\right)$$

where Z_in_ and Z_0_ are the input impedance and free-space impedance, h is the propagation constant of the microwave in material, and c is the light velocity, respectively. The refractive index n and extinction coefficient k of the porous material can be calculated by the following formula [34]:

(9) $$n^{2}=\frac{1}{2}\left(\sqrt{{\left(\varepsilon_{\varepsilon }^{\prime }\right)}^{2}+{\left(\varepsilon_{\varepsilon }^{\prime \prime }\right)}^{2}}+\varepsilon_{{}_{\varepsilon }}^{\prime }\right)$$

(10) $$k^{2}=\frac{1}{2}\left(\sqrt{{\left(\varepsilon_{\varepsilon }^{\prime }\right)}^{2}+{\left(\varepsilon_{\varepsilon }^{\prime \prime }\right)}^{2}}-\varepsilon_{{}_{\varepsilon }}^{\prime }\right)$$

When the electromagnetic wave is perpendicular to the surface of material, the reflectivity R can be expressed as:

(11) $$R=1-\frac{4n}{{\left(n+1\right)}^{2}+k^{2}}$$

With the increase in porosity, the effective permittivity and reflectivity of porous materials decrease; the greater the porosity, the more obvious the decrease, which widens the low valley of the reflection band, that is to say, the absorbing bandwidth increases significantly.

## 4. Discussion

**Figure 9:**
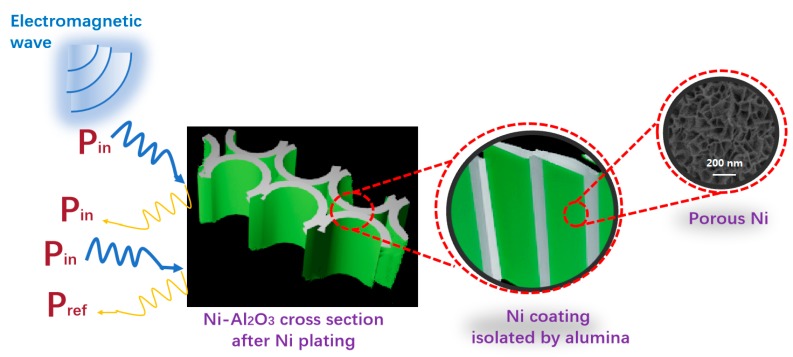
Schematic for the wave absorption mechanisms of Ni/Al_2_O_3_/Ni film cross-section after nickel plating.

Overall, the microwave absorption of the flower-like Ni/Al_2_O_3_/Ni film sample was mainly due to the presence of porous Ni, and we think that Al_2_O_3_ acted as an insulating phase to block the interaction of Ni particles and avoid the eddy current effect (Figure 9). However, in an ideal state, when a microwave is incident from the air or transition layer onto the interface of Ni/Al_2_O_3_/Ni absorbing layer, one part of the microwave reflects and the other part enters the absorber. When the input impedance of a connected network Ni/Al_2_O_3_/Ni matches the impedance of the air, no reflection of the microwaves occurs on the interface, and all of them enter the absorbing layer medium. In addition, the Ni particles are much smaller than the skin depth and the microwave wavelength. Therefore, ultrafine particles are uniformly magnetized by external alternating microwave fields, and wave energy is dissipated by hysteresis loss, eddy current loss, and other loss mechanisms [35]. Notably, the impurities, the defects, and the interactions between electrons and electrons may all be the reasons for the several tiny impurity absorption peaks in the microwave band [36]. When the microwave is incident, the 3D network structure inside the porous Ni/Al_2_O_3_/Ni can be seen as many connected tubular loops, the cut-off grid is like a microwave receiving antenna for the adjacent tubular loops, and polarized current generated under the action of high-frequency magnetic fields propagates from the network surface to inside along the staggered tubular loops rapidly [37]. According to the measurement results of microwave parameters, this propagation is attenuated due to ohmic loss due to the different directions of each tubular loop inside the porous Ni/Al_2_O_3_/Ni. The induced fields generated by the polarized current flowing through the tubular loop mostly canceled each other out and resulted in the attenuation of microwave energy. Simultaneously, the incident microwave was scattered and reflected in all directions and resulted in mutual cancellation of microwave energy of opposite directions. Similar to the transmission behavior of microwave in chiral media, most of the incident microwave was lost in the Ni/Al_2_O_3_/Ni 3D network.

## 5. Conclusions

In this study, two interconnected network Ni/Al_2_O_3_/Ni films were fabricated using controllable electrodeposition, their morphology and static magnetic properties were investigated, and the results indicated that the two connected network Ni/Al_2_O_3_/Ni films both have low permittivity, low permeability, low magnetic loss, low dielectric loss, and good microwave absorption properties under an applied magnetic field within a frequency range of 2–18 GHz. The 3D connection network loop inside the composite membrane enhances the cancellation of microwaves, the eddy current can be isolated by Al_2_O_3_ insulating medium, and the temperature generated by eddy current effect can be reduced. The XRD of the metallic Ni demonstrated wide peaks due to the nanoscale effect. Such advances were gained by controlled electrochemical deposition allowing the regulation of hole diameters of the Al_2_O_3_ scaffold nanostructure.

## Figures and Tables

**Table 1:** EDS pattern of as-prepared flower-like Ni/Al_2_O_3_/Ni film.

Element	Atomic Number	Unnormalization (wt %)	Normalization (wt %)	Atomic Percentage (at %)	Error (wt %)
**Al**	13	35.81	52.24	66.92	5.7
**O**	8	27.41	39.98	30.37	5.4
**Ni**	28	5.33	7.7	2.71	0.4

**Table 2:** EDS pattern of as-prepared grain-like Ni/Al_2_O_3_/Ni film.

Element	Atomic Number	Unnormalization (wt %)	Normalization (wt %)	Atomic Percentage (at %)	Error (wt %)
**Al**	13	33.89	49.50	39.05	1.7
**O**	8	30.15	44.05	58.61	1.4
**Ni**	28	4.42	6.45	2.34	0.4
